# Supplementary material for: Comparison of PSA Values from capillary dried blood spot analysis and venous serum samples
Source: PLoS One. 2025 Oct 29;20(10):e0335448. doi: 10.1371/journal.pone.0335448 (PMC12571311; doi:10.1371/journal.pone.0335448)
Supplement: Attachment — Submitted filename: PLOS ONE - Response to Reviewers.docx [file pone.0335448.s002.docx]

Dear Reviewers,

We would like to sincerely thank you for the thorough and thoughtful evaluation of our manuscript. Your constructive comments and valuable suggestions have helped us to improve the clarity, accuracy, and overall quality of the paper. Below, we provide detailed responses to each comment and describe the corresponding revisions made in the manuscript.

**Reviewer #1:**

The article Comparison of PSA Values from Capillary Dried Blood Spot Analysis and Venous Serum Samples is well written and clearly written, with a well-described methodology and clear conclusions. It describes the use of a new methodology for PSA analysis from capillary blood samples. This is a prospective study conducted on a group of 224 male patients. The primary data, only of total PSA, are available. The discussion is factual and the authors discuss the advantages of capillary blood samples but also mention the limitations of the method and study.

**Response:**

Thank you very much for this positive assessment and for summarizing our work so clearly.

**Reviewer #1:**

I have only one comment on the Conclusion.

I disagree with the authors' conclusion that the analysis of free PSA from DBS samples is a promising approach to improve the diagnostic specificity of the PSA value. The authors correctly state in the discussion the general and known problems in determining PSA. It is generally known that freePSA increases the sensitivity and specificity of the determination of total PSA alone. However, in line 250 the authors state that although the present study design did not allow direct assessment of retest reliability...It is therefore not possible to objectively evaluate the analytical performance of both methods used. From personal experience, I would assume that the analytical performance of the established and long-standing Roche ECLIA method will be better than methods based on capillary dried blood spots. (But I could be wrong.) In any case, without analytical performance, the claim of improved PSA specificity when using free PSA specifically from DBS samples is irrelevant. After resolving my comment and minor revision, I recommend the article for publication.

**Response:**

We appreciate this important and well-taken comment. Based on your feedback, we have revised the conclusion to more accurately reflect the scope of our findings and to avoid overinterpretation. The revised text now reads:

**Revised text:**

The analysis of free PSA from DBS samples demonstrates the feasibility of determining both total and free PSA and thereby enables calculation of the fPSA/tPSA ratio, which has the potential to improve diagnostic specificity. However, further studies are needed to confirm the analytical performance of this approach.

**Reviewer #2:**

Dear Authors,

Your study strongly demonstrates the feasibility of capillary blood samples for PSA testing. The ability to calculate the fPSA/tPSA ratio using DBS is particularly valuable for its potential to increase diagnostic specificity. However, the study requires some minor revisions.

**Response:**

Thank you for this kind introduction and positive feedback.

**Reviewer #2-1:**

Capillary samples were collected by healthcare personnel, so the true "self-sampling" scenario was not evaluated. This should be more clearly emphasized as a limitation.

**Response:**

Thank you for highlighting this important point. We have clarified this limitation in the Discussion section to better delineate the scope of the present study. The revised text reads as follows:

**Original text in the Limitations (Discussion):**

“It is important to mention that this study has some limitations. In this study, capillary blood samples were collected by trained medical professionals. This means that pre-analytical errors, which are possible when capillary blood samples are taken by the patients themselves, were not recorded. Therefore, no conclusive statements can be made regarding its use outside the medical environment.”

**Revised text:**

It is important to mention that this study has some limitations. Capillary blood samples were collected exclusively by trained medical professionals under controlled conditions. Therefore, the results primarily demonstrate feasibility in a clinical setting, but they do not directly reflect a true self-sampling scenario. Pre-analytical errors that may occur during patient-performed self-collection were not captured, and the transferability of these findings to unsupervised home-based sampling remains uncertain. Future studies should explicitly investigate the reliability and accuracy of patient self-sampling.

**Reviewer #2-2:**

High PSA values (>15 ng/ml) were excluded from the study; the clinical impact of this condition should be further investigated. It is recommended that these limitations be addressed more clearly and the Discussion section be strengthened accordingly.

**Response:**

We agree with this valuable suggestion. We have expanded the Discussion to emphasize the clinical relevance of this limitation. The revised paragraph reads:

**Original text (Discussion, current):**

“Although greater variability (up to approximately 30%) was observed in higher PSA ranges (>10 ng/ml), the number of cases was very limited (n=4), restricting reliable conclusions. Such deviations align with known methodological and biological variabilities of PSA testing, and are clinically less relevant since elevated PSA-values results typically require confirmatory venous measurements and further diagnostic evaluation (15, 16). Therefore, deviations in the higher range are of limited clinical relevance, as the primary function of the initial PSA screening - identifying patients requiring further evaluation - remains preserved.”

**Revised text:**

Although greater variability (up to approximately 30%) was observed in higher PSA ranges (>10 ng/ml), the number of cases was very limited (n=4), restricting reliable conclusions. Such deviations align with known methodological and biological variabilities of PSA testing, and are clinically less relevant since elevated PSA values typically require confirmatory venous measurements and further diagnostic evaluation (15, 16). Nevertheless, the exclusion of patients with very high PSA values (>15 ng/ml) represents an important limitation, as this subgroup is of particular clinical relevance for prostate cancer diagnostics and treatment decisions. The diagnostic performance of capillary DBS sampling in this range could not be adequately assessed in this study and should therefore be investigated in larger patient cohorts in future research.

**Closing Statement**

We hope that our revisions and clarifications adequately address all reviewer comments. We are grateful for the reviewers’ and editor’s constructive feedback, which has helped us significantly improve the manuscript. We hope that the revised version meets the journal’s publication standards and look forward to your favorable consideration.

Thank you for your time and consideration.

With best regards,

Lucas Engelage,

on behalf of all authors
